# Health system influences on the implementation of tuberculosis infection prevention and control at health facilities in low-income and middle-income countries: a scoping review

**DOI:** 10.1136/bmjgh-2020-004735

**Published:** 2021-05-11

**Authors:** Gimenne Zwama, Karin Diaconu, Anna S Voce, Fiona O'May, Alison D Grant, Karina Kielmann

**Affiliations:** 1 Institute for Global Health and Development, School of Health Sciences, Queen Margaret University, Edinburgh, UK; 2 Public Health Medicine, School of Nursing and Public Health, University of KwaZulu-Natal, Durban, South Africa; 3 TB Centre, London School of Hygiene & Tropical Medicine, London, UK; 4 Africa Health Research Institute, School of Laboratory Medicine & Medical Sciences, College of Health Sciences, University of KwaZulu-Natal, Durban, South Africa; 5 School of Public Health, University of the Witwatersrand, Johannesburg, South Africa

**Keywords:** tuberculosis, review, health systems evaluation, prevention strategies

## Abstract

**Background:**

Tuberculosis infection prevention and control (TB-IPC) measures are consistently reported to be poorly implemented globally. TB-IPC guidelines provide limited recognition of the complexities of implementing TB-IPC within routine health systems, particularly those facing substantive resource constraints. This scoping review maps documented system influences on TB-IPC implementation in health facilities of low/middle-income countries (LMICs).

**Methods:**

We conducted a systematic search of empirical research published before July 2018 and included studies reporting TB-IPC implementation at health facility level in LMICs. Bibliometric data and narratives describing health system influences on TB-IPC implementation were extracted following established methodological frameworks for conducting scoping reviews. A best-fit framework synthesis was applied in which extracted data were deductively coded against an existing health policy and systems research framework, distinguishing between social and political context, policy decisions, and system hardware (eg, information systems, human resources, service infrastructure) and software (ideas and interests, relationships and power, values and norms).

**Results:**

Of 1156 unique search results, we retained 77 studies; two-thirds were conducted in sub-Saharan Africa, with more than half located in South Africa. Notable sociopolitical and policy influences impacting on TB-IPC implementation include stigma against TB and the availability of facility-specific TB-IPC policies, respectively. Hardware influences on TB-IPC implementation referred to availability, knowledge and educational development of staff, timeliness of service delivery, availability of equipment, such as respirators and masks, space for patient separation, funding, and TB-IPC information, education and communication materials and tools. Commonly reported health system software influences were workplace values and established practices, staff agency, TB risk perceptions and fears as well as staff attitudes towards TB-IPC.

**Conclusion:**

TB-IPC is critically dependent on health system factors. This review identified the health system factors and health system research gaps that can be considered in a whole system approach to strengthen TB-IPC practices at facility levels in LMICs.

**Key questions**

**What is already known?**

- Health facilities in settings with high tuberculosis (TB) prevalence are places where people are exposed to a high risk of TB transmission.
- Implementation of TB infection prevention and control (TB-IPC) measures at health facilities in low/middle-income countries (LMICs) is suboptimal.
- TB-IPC guidelines do not adequately consider the health system-related challenges to TB-IPC implementation.

**What are the new findings?**

- We mapped four broad health system domains that influence implementation of TB-IPC at facility level in LMICs including hardware, software, policy and decisions, and contextual factors.
- The most commonly noted influences within each of these system domains were, respectively, human resources, norms and values, availability of policies and guidelines, and TB stigma.
- There are substantial knowledge gaps in understanding systemic cross-cutting influences and interactions that have bearing on sustainable TB-IPC implementation, partly explained by limitations in research design and analyses.

**What do the new findings imply?**

- TB-IPC implementation is critically dependent on health system influences.
- For optimal TB-IPC, whole system approaches are necessary to understand the complexities surrounding TB-IPC implementation and to inform the development of appropriate policy and strategies for strengthening TB-IPC practices.

## Introduction

Tuberculosis (TB), including drug-resistant TB (DR-TB), remains the world’s leading infectious disease challenge, accounting for 1.5 million deaths yearly and over one in four deaths (29%) attributable to antimicrobial resistance.^1 2^ The risk of transmission is high in congregate settings, including health facilities.^3^ Health facilities are also likely to host high numbers of people with undiagnosed active TB,^4^ and hence pose a high TB transmission risk for healthcare providers,^5–7^ and potentially patients.^8^ In low/middle-income countries (LMICs), where TB incidence is high,^1^ implementation of TB infection prevention and control (IPC) measures to reduce the risk of transmission of TB infection in health facilities is consistently reported to be poor.^9–12^ Poor healthcare provider knowledge and motivation as well as poor infrastructure and personal respiratory protection supplies are common explanations provided for suboptimal implementation.^9 10 13–17^

The WHO published the latest iteration of TB-specific guidelines for the prevention and control of transmission of TB infection in health facilities in 2019.^18^ The guidelines recommend a hierarchy of three broad types of measures that need to be implemented for successful TB-IPC in health facilities: administrative controls, environmental controls and respiratory protection. The guidelines acknowledge an interplay between the implementation of these measures and health system functioning by referring to core components of broader IPC programmes. Core components are noted to relate to national and facility level and include: (1) guidelines; (2) education and training; (3) healthcare-associated infection surveillance; (4) multimodal strategies including system and culture change; (5) monitoring and feedback; (6) as well as facility-level resources of (7) workload, staffing and bed occupancy; and (8) the built environment, materials and equipment.^19^

Despite appeals for holistic strategies,^19–22^ guidelines for TB-IPC do not adequately account for the complexity of the health system contexts within which they are interpreted and operationalised. Effective implementation relies on both the tailoring of measures to specific health facility contexts as well as wider health system institutional, behavioural and organisational factors,^23^ such as variations in facility design, management practices and patient load. The lack of consideration of these factors may reflect gaps in the ways in which TB-IPC implementation has been investigated at health facility level.

This scoping review aims to characterise studies of TB-IPC implementation at health facility level in LMICs and map the documented health system influences on TB-IPC implementation.

## Methods

### Design

A scoping review methodology was deemed appropriate to investigate health system influences on TB-IPC implementation at health facilities in LMICs as relatively little is known about this complex and discursive subject. Our purpose was to map key concepts, types of evidence and gaps in research on this subject.^24^ We followed the processes for undertaking scoping reviews as recommended by Arksey and O’Malley, and Levac *et al*.^24–26^ Accordingly, we present our research question (stage 1), the search strategy used to identify relevant literature (stage 2), study selection (stage 3), data extraction processes (stage 4), and collating, summarising and reporting of results (stage 5). In stage 5, we drew on best-fit framework synthesis methods and charted the extracted data against the health policy and systems research framework proposed by Sheikh *et al* (online supplemental figure).^27 28^

10.1136/bmjgh-2020-004735.supp1 Supplementary data

### Research question

This review was guided by the question: What health system influences on TB-IPC implementation in LMIC health facilities have been assessed in the existing literature?

### Search strategy

On 4 July 2018, we searched PubMed, CINAHL plus with full text (via EBSCOhost), Medline (via EBSCOhost), Web of Science and Scopus without applying language and publication date search limitations. Search terms included synonyms and Medical Subject Headings terms of tuberculosis, nosocomial transmission and infection prevention and control (online supplemental table 1).

### Study selection

The full list of final inclusion and exclusion criteria is available in online supplemental table 2. With particular interest in countries where systems may face financial resource constraints in responding to a high TB burden, we included primary research studies conducted in LMICs describing TB-IPC implementation processes or practices at health facility level. LMICs were defined as countries with a gross national income lower than $12.375 per capita, as per World Bank calculations for 2020.^29^ We included articles that reported on TB-IPC measures as described by the WHO in 2009.^3^

We imported all database results into Mendeley Referencing software and removed obvious duplicates. Subsequently, we transferred all titles and abstracts of the remaining search results to Rayyan QCRI to facilitate screening of titles and abstracts. We manually removed any remaining duplicates and marked individual entries with labels indicating the reasons for inclusion or exclusion. We further examined full-text versions when the title and abstract were insufficient to determine eligibility. Reviewers GZ, FO’M and KK screened 69.5%, 42.5% and 10% of search results, respectively, double screening nearly one-third of documents (31%). We noted a high level of agreement (first round of double screening: 85%; second round: 99%) and resolved any disagreements by consensus.

### Data extraction

The data extraction form was developed jointly by two reviewers (GZ and FO’M) and piloted on a subset of included studies, then refined prior to use across all studies (see online supplemental file 2). We extracted bibliometric information (author, title, publication year) and information on study aim, type, setting, methodology, methods, study participants and health system influences. Informed by the WHO’s definition of a health system,^30^ we adopted a deliberately broad perspective and extracted all information from the Results sections that described any contextual and health system influences, including system actor characteristics surrounding TB-IPC implementation at health facility level. One reviewer (GZ) extracted bibliographical data as well as direct textual quotations or, where possible, descriptive summaries describing any investigated or reported influences on TB-IPC implementation at health facility level. Data extraction was conducted iteratively: all papers were read twice to ensure they capture all relevant information.

10.1136/bmjgh-2020-004735.supp2 Supplementary data

### Collating and summarising

Following data extraction, we used a stepwise approach to analysis. First, we conducted a bibliometric summary of study characteristics. Second, in line with the principles of a best-fit framework synthesis, we explored potential frameworks with components that a priori were relevant to our study objective (ie, mapping health system influences on TB-IPC implementation at facility level).^27^ We adopted the health policy and systems research framework proposed by Sheikh *et al*^28^ (see online supplemental figure), and as elaborated by Gilson.^31^ The Sheikh *et al* framework incorporates the perspective that health system ‘hardware’ and ‘software’ interact and that health policy and systems are shaped by and within the surrounding social and political contexts. This perspective provided an overarching coding framework that guided the categorisation of previously extracted data on whole system influences (table 1). Two reviewers (GZ and KD) reviewed the extracted quotes or summaries and deductively coded information against this coding framework.

**Table 1:** Coding framework

Element	Item	Definition
**Social and political context**		Broader social and political environment, discourse and norms that shape policy decisions and the structure, organisation and practice within health systems, for example, social stigmatisation and wider political priorities.
**Policy and decisions**	**Health policy**	‘Health policy is commonly seen as the formal written documents, rules and guidelines that present policymakers’ decisions about what actions are deemed legitimate and necessary to strengthen the health system and improve health,’^28^ for example, availability and content of TB-IPC guidelines at national or facility level.
	**Policy decisions**	‘The processes of decision-making at all levels of the health system and the wider influences that underpin the prioritisation of policy issues, the formulation of policy, the processes of bringing them alive in practice and their evaluation’,^28^ for example, the translation of policy into formal programmes.
**Health system hardware**	**Human resources**	Availability and types of health workforce and aspects of human resource capacity (eg, TB-IPC knowledge, skills and training).
	**Organisational structure**	Governance structures, including logistics, coordination, support and supervision systems. Procedures and processes of care, forms of service delivery, routines, allocation and management of responsibilities and demand.
	**Medicine and technology**	Availability of medications, for example, isoniazid preventive therapy and TB medication. Availability and functionality of medical devices and equipment, for example, respirators, surgical masks, extractor fans and ultraviolet germicidal irradiation.
	**Service infrastructure**	Physical infrastructure, including space, its layout and ventilation. Technologies targeting augmentation of physical environment (eg, whirlybirds, retrofits).
	**Information systems**	Availability of patient information systems (eg, record systems). Information dissemination, communication and reporting structures/mechanisms, flows and ways in which these are constructed.
	**Financing**	Any financing implications affecting system hardware (eg, budget allocation for equipment) and software (eg, incentives).
**Health system software**	**Values and norms**	System actors’ priorities. Accepted practices and established ways of behaviour of patients at health facility level and healthcare providers across the system, workplace culture. Professional identity and cultural beliefs and perceptions of actors in the system.
	**Relationships and power**	Constellation and communication of actors in the system, their inter-relation and relative power over relationships, hardware and policy space (eg, authority, autonomy, issues of legitimacy).
	**Ideas and interests**	Expectations, motivation, willingness and satisfaction of actors in system.

TB-IPC, tuberculosis infection prevention and control.

### Reporting

Given that the purpose of this review was to identify and map key influences on TB-IPC implementation, and that in most cases this information was not an explicit objective of included studies but rather part of observational and analytical accounts offered by study authors, we did not quality assess included studies. We followed Preferred Reporting Items for Systematic Reviews and Meta-Analyses extension for scoping reviews reporting guidelines.

### Patient and public involvement

Patients or the public were not involved in the design, or conduct, or reporting, or dissemination plans of our research.

## Results

**Figure 1:**
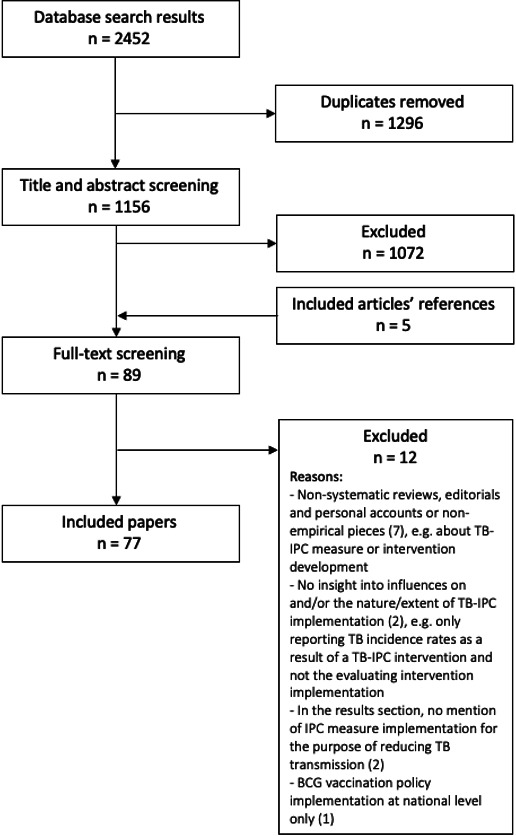
Flow diagram: summary of papers retrieved, screened and included. TB-IPC, tuberculosis infection prevention and control.

Figure 1 presents a flow chart of the outcome of the search and screening process. The electronic database search identified 2452 publications; 1156 unique entries were retrieved of which titles and abstracts were screened. We perused the full texts of 84 publications and identified 5 additional relevant papers through screening of reference lists. After reading the full texts, our review included 77 publications.

### Study characteristics

#### Regional focus

**Figure 2:**
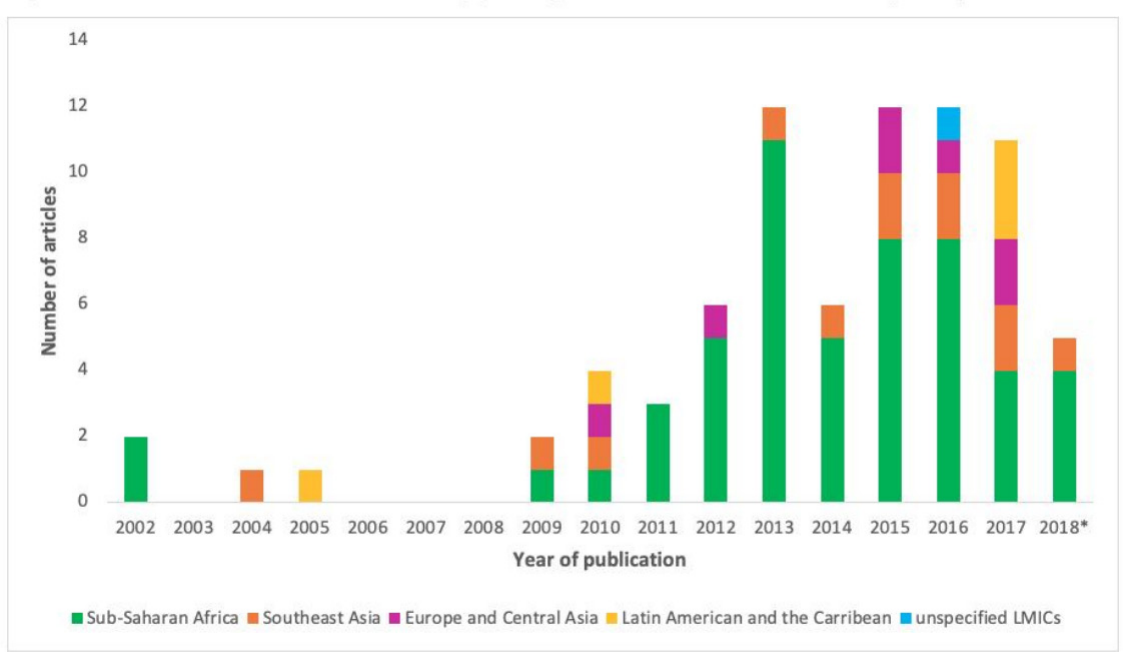
Distribution of number of articles by year of publication. *Published before July 2018. LMICs, low/middle-income countries.

Published empirical pieces of research documenting TB-IPC implementation at health facilities in LMICs were very few pre-2008 (n=4, 5.2%),^32–35^ but increased post-2008 (figure 2). Just over two-thirds of the studies were conducted in sub-Saharan Africa (n=52, 67.5%),^5 11 16 17 32 34 36–81^ and more than half of these were in South Africa (n=28, 53.8%).^5 16 17 34 58–81^ Twelve studies were conducted in Southeast Asia (15.6%),^14 33 82–91^ seven in Europe or Central Asia (9.1%),^15 92–97^ and five in Latin America and the Caribbean (6.5%).^35 98–101^ One study adopted a general LMIC focus in which the country remained unspecified (1.3%).^102^

#### Level of care

Just over half of all studies (n=41, 53.2%) investigated TB-IPC implementation at hospitals, including district, regional, secondary or tertiary care levels.^11 14 16 32 33 35 48 49 54 55 58–61 64 66 70–80 82–87 90–92 96–100^ Twelve studies (15.6%) focused on facilities at primary care level,^5 17 34 38 43 53 62 63 65 67 81 101^ and 19 (24.7%) on a variety of facilities at primary and higher care levels.^15 36 37 39–41 44 45 50–52 57 68 69 88 89 93–95^ Five studies (6.5%) did not provide sufficient detail on the level of health facility investigated.^42 46 47 56 102^

#### Participants

Roughly three-quarters of all included articles (n=59, 76.6%) reported on TB-IPC implementation from a provider point of view.^5 11 15 16 32–34 36 37 39 40 42–47 49 50 53–58 60 62–64 66 68 70 72 74–83 85–87 89–101^ Eight studies (10.4%) included patient perspectives,^32 39 41 56 65 67 82 85^ of which three (3.9%) reported on TB-IPC implementation from a patient perspective exclusively.^41 65 67^ One-fifth of studies (n=15, 19.5%) did not adopt either a patient or provider perspective, but rather more generally evaluated or assessed TB-IPC implementation at facility level.^14 17 35 38 48 51 52 59 61 69 71 73 84 88 102^

#### Intervention studies

Thirteen intervention studies were included (15.6%).^32 42 43 57 59 68 79 87 88 93 94 97 102^ Most (n=7, 53.8%) focused on capacity building for TB-IPC implementation and evaluation,^42 43 68 79 87 93 94^ and two included training as an intervention component.^57 88^ Other studies evaluated the introduction of TB-IPC guidelines (n=2, 15.4%)^32 102^ and a screening programme (n=1, 7.7%).^57^ Four studies (30.7%) adopted a participatory approach to developing a TB-IPC plan template, a triaging protocol and accompanying referral system, a reporting mechanism and posters, respectively.^59 93 94 97^ Three studies (23.1%) adopted longer term evaluation cycles or mentoring for the implementation of TB-IPC interventions.^42 68 88^

#### Data collection methods

Three-quarters of the studies (n=58; 75.3%) used structured data collection tools including questionnaires, checklists, audit and assessment tools, and record extraction, which were also used for structured interviews and observations.^5 11 14–17 32–35 37 38 40–47 49 50 52–56 59 62–70 72 75 77–79 82 84–95 97 100 102^ Thirty studies (40.0%) adopted observations.^5 14 17 37 40 42–45 47 50 52 53 56 59 61–64 66 68 69 71 72 75 83 84 86 88 102^ One study additionally conducted a patient waiting survey,^17^ and another followed patients through the facility.^50^ Thirteen studies (16.9%) implemented group discussion methods,^36 39 40 47 60 75 79–81 96 99 101^ and 17 studies (22.1%) used semistructured or in-depth interviews.^39 43 44 53 56–58 60 68 72 74 80 82 83 86 98 99^ Document-based data were extracted from patient, employee or facility-level records as well as facility-level documents and policies (n=18, 23.4%).^5 17 32 35 38 40 42 43 48 50 51 59 64 72 73 75 78 100^ One study reviewed national policy documents (1.3%).^83^ Other data collection methods included diagnostic testing (n=3, 3.9%),^14 34 100^ air microbacteria sampling (n=3, 3.9%),^33 71 87^ as well as ventilation measurements (n=6, 3.9%).^17 33 40 47 88 100^

### Health system influences on TB-IPC implementation

None of the studies set out to primarily investigate wider system or health system influences on TB-IPC implementation. However, all papers presented some assessment or narrative account of influences on TB-IPC implementation. Extracted information on health system influences was coded deductively against the health policy and systems framework by Sheikh *et al*^28^ as further defined in the online supplemental figure and adapted in table 1. In online supplemental table 3 and below, we describe the influences categorised as social and political context, policy decisions, health system hardware and software, as well as influences on TB-IPC beyond this framework.

#### Social and political context

A little more than half of the studies (n=42; 54.5%) illustrated the influence of the overarching context on TB-IPC implementation at health facility level, either directly or indirectly, by influencing other identified influences in this review, or by affecting the number and infectiousness of patients with TB at health facility level. The least frequently noted influence was that of political relationships, interests and agendas (n=2),^83 98^ this included reference to China’s growing economic influence on occupational health regulations.^83^ Social influences more directly influencing TB-IPC implementation at health facility level included stigmatisation of patients with TB (n=9)^40 47 60 67 74 77 81 93 98^ and community awareness of prevention measures and TB (n=5).^39 67 74 82 85^ The influence of social capital on healthcare provider TB-IPC implementation or adherence to care of patients with TB reflected the importance of support systems (n=4).^36 66 77 98^ For instance, healthcare providers’ fear of losing their job if they were to acquire multiple or extensive DR-TB, identified as an influence under ‘ideas and interests’, was rooted in concerns about their family’s financial well-being.^77^ Patient poverty-related issues, such as job insecurity and a lack of transport money to go to the health facility, were also reported (n=3).^36 74 98^ From a providers’ perspective, the availability of a social grant for poor and moribund patients with TB negatively influenced treatment adherence.^74^ Few studies noted the influence of patient family values and norms on TB-IPC implementation (n=2),^36 98^ and of traditional medicine and self-medication resulting in the late uptake of care (n=2).^74 99^ Different examined contextual influences on TB-IPC implementation at health facility level included patient health or demographic profile (n=9),^35 38 39 41 46 50 65 67 72^ as well as influences inherent to facility location such as geographical variations (n=14),^5 40 41 51 53 56 63 67 69 83 84 90 92 96^ and seasonal or circadian weather variations (n=12).^14 16 17 43 53 61 71 72 77 80 81 96^

#### Policy decisions

Thirty-four studies (44.2%) reported on the influence of policy decisions on TB-IPC implementation, including health policy (n=32) and policy processes (n=26).

Studies reporting on health policy particularly focused on the availability of facility-level TB-IPC,^40 43 47 50 62 64 69 84 86 90 91 93 94 102^ or IPC plans (n=21).^5 17 36 37 42 72 88^ Researchers paid considerable attention to the availability of national policies and guidelines (n=14).^16 37 42 43 49 50 74 78–81 91 93 94^ Of these studies, only few considered the applicability of national policy to facility-level implementation of TB-IPC.^15 81 83^ Studies also reported the availability of more specific regulations (n=13),^5 15 17 42 50 63 64 78 82 84 91 93 102^ such as for masks,^5 17 102^ visitors^64 82^ and waiting time monitoring.^42^ Occupational health regulations surfaced in a small number of studies (n=5),^5 17 78 79 83 94^ with more specific attention to history^83^ and scope^83 94^ thereof.

Reported policy processes encompassed the influence of policy decision-making, translation and evaluation processes on TB-IPC implementation. Numerous studies looked at whether TB-IPC practices were monitored and evaluated (n=17),^5 16 36 42 43 47 62 63 78–81 83 84 88 91 93^ with some paying attention to the frequency thereof (n=7).^42 43 78 84 88 91 93^ Studies that considered quality improvement processes were either intervention studies or specifically referred to improvements of clinic layout and ventilation (n=10).^42 43 59 68 79 86 88 91 93 102^ Stakeholders involved in policy development processes were healthcare providers, IPC committee members and experts (n=7).^42 59 81 83 87 90 94^ One study noted the influence of the involved stakeholders’ degree of authority.^83^ Lastly, processes of policy accountability were investigated in only seven studies.^32 42 43 52 80 81 83^ This included healthcare providers’ perceived inability to prove occupationally acquired TB in high-endemic countries (‘TB is everywhere’).^80 81^

#### Health system ‘hardware’

All 77 studies reported on hardware influences, predominantly human resources (n=66), organisational structure (n=61) as well as medicine and technology (n=52). Service infrastructure (n=46), that is, the physical infrastructure of facility space and its augmentation, information systems (n=31) and financing (n=27) were less investigated.

#### Human resources

Pertinent human resource influences on TB-IPC implementation included healthcare provider and health service users’ educational development (n=45) and knowledge (n=40), as well as the availability of staff (n=36). Furthermore, studies examined the influence of healthcare provider demographics (n=19),^15 36 44 45 49 50 53–55 57 60 63 64 70 79 80 89 92 96^ for example, job category and years of experiences, and their own or their colleagues’ health (n=5)^5 58 66 81 96^ on their TB-IPC implementation practices.

Commonly identified educational influences were the availability of or participation in staff (TB-)IPC training (n=37)^36 41 43 44 47 49 50 52 53 55 60 62–64 70 72 74 79–82 84 86–89 93 94 96 98 101^ and the education of communities on TB, IPC or health (n=21).^32 36 37 39 43 44 47 52 53 62 67 69 72 80–82 84 91 93 98 100^ Studies also highlighted training characteristics, that is, type of training,^17 33 36 42 43 58 70 72 74 79 81 82 84 88 89 91 93 94 96 98^ target,^36 37 43 64 72 74 79 81 86 88 91 93 94 98 101^ frequency,^37 43 54 55 62–64 72 81 88 91 93 96 98^ duration^33 54 93^ and adequacy.^64^ Few training efforts focused on staff capacity to address daily challenges at facility level or to engage in reflective practice for improvement.^33 42 79 81^

Staff knowledge of TB-IPC measures was a primary topic of investigation (n=28),^11 16 40 42 44 49 53 54 59 62–64 66 68 72 74 75 81–83 85 89 93–96 101^ followed by staff knowledge on TB infection, disease and treatment (n=16).^11 15 16 44 55 58 64 66 68 74 89 91 95 96 98 99^ Publications presented considerable attention to staff knowledge of TB transmission (n=11),^11 15 16 32 55 58 66 74 89 95 96^ IPC-related policies (n=9)^16 53 68 70 74 80 81 87 99^ and TB risk factors, respectively (n=7).^11 15 50 55 66 86 96^ Some studies also examined knowledge of TB risk (n=5).^15 58 59 85 99^ Individual studies reported staff knowledge of epidemiology,^93 99^ patient context,^98^ or research/programme implementation and computer skills.^68^

Studies commonly referred to the availability of staff with responsibilities for IPC (n=19),^16 17 40 42–44 50 51 53 57 58 64 69 76 78 84 88 93 102^ and in some cases TB-IPC (n=7).^43 52 69 72 76 87 90^ Few studies noted the influence of IPC focal persons’ qualification,^50 58 78^ time^64 78^ and continuity.^88^ More studies noted the influence of general staff availability on TB-IPC implementation (n=14),^43 47 49 53 58 74 79–82 91 93 94 99^ and, to a lesser extent, staff turnover.^57 82 91^ Further, studies reported on the availability of staff with specific roles (n=14),^5 17 32 42 43 53 62 64 78 81 82 88 91 98^ such as an occupational health nurse,^64 78^ dedicated staff to open windows^62 81 88^ and environmental control engineering staff.^5 53 88 91^

#### Organisational structure

Many studies investigated or reported on the coordination and timeliness of diagnostics and service delivery (n=33), encompassing the timeliness and coordination of patient fast-tracking, triaging and separation as well as turnaround times of, and subsequent action on, laboratory results.^16 17 32 34 35 39–43 47 50 52 53 59–62 66 68 72–74 79–82 91 92 94 98 100 101^ Studies considered the influence of the allocation, uptake and governance of health system actors’ demands and responsibilities on TB-IPC implementation (n=27).^36 40 43 47 49 50 53 57 58 60 62 66 68 74 76 78–83 88 91 93 94 96 98^ A comparable number reported on the influence of occupational health systems and support on the implementation of TB-IPC (n=26).^14 34 36 42–44 47 50 51 57 58 64 66 69 72 77 78 80 81 83 84 88 91 93 94 98^ Studies focused on the timing or point at which TB-IPC measures were implemented, for example, at facility entry, and at whom these were targeted (n=19).^16 37 40 43 44 47 50 53 55 62 64 66 69 72 74 80 82 99 100^ Further, attention was paid to the influence of variations between different facilities (n=18), comparing facilities as a whole,^16 38 53 76 78 96^ level of care,^15 40 50–52 57 84 90 92^ facility ownership^40 41 47 50–52^ and services on offer.^15 42 51 52^ Management of space (n=17) included the influence of the use of space for multiple purposes, the allocation of spaces to specific services and their location as well as the sharing of spaces by patients with different morbidities, and overcrowding of spaces.^14 17 36 40 47 50 53 72 74 75 77 79–81 96 98 101^ The influence of facility usage and service delivery demand arose from 12 studies.^17 38 40 50 52 77 83 84 91 92 98 99^ Studies also examined the existence of IPC-related committees (n=11),^17 36 47 58 62 72 76 78 82 84 93^ with specific attention to committee meetings,^17 62 72 82 88^ make up,^72 82^ allocated functions^76 82^ and budget.^72^ Factors associated with the alternative management of exposure to infectious patients with TB (n=11) included influences such as healthcare provider time spent in contact with patients with TB, movement and ward transfers of patients with TB.^32 37 58 64 71 73–75 80 89 100^

#### Medicine and technology

Medicine and technology influences predominantly referred to the availability of masks and respirators to service users and staff (n=40).^5 14–17 36 40 42–44 47 49 52 53 55 60 62–64 66 69 70 72 74 75 77 79–82 84 86 88 91 93 96 98–100 102^ This was followed by respirator functionality (n=24)^11 16 17 36 37 42 49 53 58 60 63 64 66 72 77 78 80 81 84 93 96 98 99 102^ and the availability of engineering controls, including ultraviolet germicidal irradiation and mechanical ventilation (n=25).^16 36 42–44 47 49 50 53 56 58 62 64 69 71 72 74 77 80 81 83 84 92 94 102^ In terms of respirator functionality, studies noted the influence of quality,^64 78 93 96^ cost^80 96 98 99^ and maintenance on wearing practices.^37 96^ More commonly, studies examined possibilities for fit testing,^17 42 62 64 72 80 81 84 93 96 102^ for example, the availability of fit-testing kits or different mask types and sizes, and the influence of respirator design.^11 16 36 49 53 58 60 64 66 77 80 81 96^ Studies referred to the availability of disinfection and waste disposal materials (n=20),^16 40 42 43 47 49 55 61–64 66 72 75 79 81 82 88 93 102^ and the availability of personal protective equipment (n=15).^36 42 49 56 58 61 64 74 77–79 81 93 101 102^ Fewer studies examined the conduct of maintenance (n=11)^5 14 43 53 56 71 72 77 80 81 94^ and functionality of engineering controls (n=7).^63 64 72 77 80 81 94^ Least mentioned were the availability of infrastructure and technologies for TB diagnostics (n=8),^17 40 44 52 55 56 58 92^ medicines for HIV treatment or the prevention and treatment of TB (n=4),^42 44 55 72^ side effects and TB treatment course duration (n=2),^74 98^ and equipment to keep patients warm while windows are open (n=1).^66^

#### Service infrastructure

Most predominantly, studies investigated the availability of space for separation of patients with (presumptive) TB, such as waiting areas, isolation wards and sputum collection areas (n=31),^5 16 17 40 42–44 47 50 52 53 55 56 61–64 72 74 75 80 81 83 84 88 91 92 98 100–102^ or, more vaguely, the availability of adequate space (n=11).^17 36 40 47 50 56 77 79 81 94 101^ Moreover, studies emphasised the availability of infrastructure and clinic design for appropriate natural ventilation, such as cross-ventilation and outdoor spaces (n=26).^17 36 37 40 42 43 50 53 56 62 66 69 71 72 74 77 79–82 86 88 91 98 100 102^ Studies frequently noted facility-building structure as influencing TB-IPC implementation (n=22),^17 40 43 47 50 53 55 56 58 62 69 72 75 79–82 84 93 94 100 101^ with some additionally reporting on facility layout^55 58 62 69 72 75 82 84^ or modifications.^40 43 47 53 72 75 82^ Studies commonly compared TB-IPC implementation between locations within facilities, that is, specific areas, wards and departments (n=22).^14 16 17 33 37 40 44 47 50 53 61 62 64 73 74 79 82 84 87 88 96 100^ Lastly, one study noted the reliability of electricity as an influence on TB-IPC implementation.^72^

#### Information systems

Information systems mostly encompassed the influence of information, education and communication (IEC) materials and tools for TB-IPC, for example, the availability of screening checklists, ‘open windows/doors’ stickers and TB-IPC educational posters (n=21).^42 43 47 49 50 53 59 62–64 72 74 79 81–83 88 91 93 97 102^ Many studies mentioned the influence of standardised and systematic record keeping and reporting of patient and staff screening and diagnosis (n=16).^17 32 38 42 43 50 52 59 69 72 73 78 81 83 90 94^ Furthermore, studies reported on data capturing of TB-IPC implementation (n=14), for example, by means of an open-window register, IPC committee meeting minutes, or recording TB-IPC training participation and fit testing.^5 32 43 53 62 63 69 71 72 79 81 83 88^ An intervention study reported the development of an occupational health and safety information system in response to participants’ needs.^79^

#### Financing

Predominantly, studies made reference to ‘funding’ or ‘resources’ in broad terms (n=21), pointing to financing in relation to TB-IPC as an underinvestigated area.^16 38 40 47 49 53 58 62–64 66 68 79–81 88 93 94 96 98 99^ One study reported the sufficiency of finances for TB-IPC implementation at health facility level in relation to regional operations.^96^ Few studies specified funding (n=4) for maintenance^94^ or purchases of TB-IPC supportive equipment.^11 42 43^ Explorations of incentives for staff working in high-risk environments and compensation of workers with occupationally acquired TB infections were more elaborate and diverse (n=8).^58 64 77 80–83 98^ Some studies noted incentives for adherence to TB-IPC measures,^64 99^ the delivery of accurate TB-IPC or healthcare provider TB surveillance reports,^83^ and awards for excelling TB nurses.^81^

#### Health system ‘software'

In relation to software influences (n=56; 72.7%), the majority of reviewed studies discussed the influence of norms and values (n=52), followed by studies focusing on relationships and power (n=33) as well as ideas and interests (n=31) on TB-IPC implementation.

#### Norms and values

Almost half of the studies investigated the influence of correct and consistent use of TB-IPC measures at health facility level on other influences within the framework, the use of other TB-IPC measures or on TB-IPC implementation more generally (n=38).^14–17 34 37 38 40–42 47 49 50 53 55 58 61 63 64 66 68–72 74 75 77 79 80 82 84 88 93 94 98–100^ Workplace values and ways of doing included the influence of workplace HIV and TB stigma, cultural beliefs, habits and TB-IPC positive practice environments (n=24).^16 36 40 43 53 57 58 60 66 72 74 75 77–82 93 94 96 98 99 101^ Some studies highlighted the stigmatisation of nurses working in TB specialist hospitals,^80^ and another noted that inaction on TB-IPC was ascribed to a lack of clearly allocated responsibilities.^66^ Staff acceptability of and attitudes towards TB-IPC measures represented another commonly investigated influence under the norms and values category (n=22).^11 15 39 40 43 50 53 57 62–64 66 68 70 74 78–82 93 96^ Studies also reported on the perceived importance of TB-IPC by staff and patients (n=19),^15 16 36 39 50 53 55 58 65–68 80 81 94 96 97 99 101^ and patient acceptability of TB-IPC measures (n=14).^16 36 39 40 53 60 64–67 77 81 98 101^ System and facility-level priorities (n=9)^36 42 47 49 53 94 96 98 101^ included report that hospital management only invests money to improve TB-IPC in the clinic when they themselves would receive a kind of benefit from external bodies.^47^ Least reported influences were patient TB treatment non-adherence (n=4),^36 60 67 74^ for example, due to drugs and substance abuse or a lack of education on side effects, and the late uptake of hospital care by patients or guardians (n=2).^74 99^

#### Relationships and power

Commonly reported influences in this domain were agency (n=26), collegiality (n=15) and confidentiality, trust and rapport (n=15). Agency pertained to staff,^36 40 42–44 47 58 60 66 68 72 74 75 78 80 81 86 93 94 98 101^ patients,^39 98^ and IPC committees^17 82^ and encompassed coping strategies, feelings of empowerment and difficulties with TB-IPC implementation.^16 17 36 40 42 43 47 57 58 60 64 68 72 74 75 78 80–82 86 93 94 98 101^ Examples for staff included their ability to deal with patients^16^ and autonomy to enact change.^68^ The influence of collegiality among staff as well as between staff and patients consisted of elements of cooperation, collaboration and managerial support.^36 40 44 49 58 62 66 68 72 74 77 81 82 93 98^ For example, a study reported that participants were aware they needed the cooperation of patients for TB-IPC implementation.^40^ There were also shared concerns about confidentiality, trust and rapport, for example, affecting healthcare workers’ disclosure of their health status at work and care-seeking at their employing facility.^16 36 40 53 56 57 60 66 68 74 78 81 94 99^ Rapport between patients and healthcare providers was upheld by closing doors during consultations for patient privacy, thereby compromising ventilation.^36^ The role of system actors, for example, managers, district officers, non-governmental organisations and health institutions, also surfaced as influencing TB-IPC implementation (n=4).^40 47 81 98^

#### Ideas and interests

Most frequently discussed ideas and interests influencing TB-IPC implementation were patient and staff TB risk perceptions and fears (n=23).^15 16 36 39 40 49 55 58 60 63 64 66 74 77 80–83 96–99 101^ Studies recurrently reported healthcare provider or patient ideas about TB-IPC measure effectiveness (n=16),^16 36 55 58 64 67 74 77 80 81 83 96–99 101^ and considered the influence of healthcare provider motivation, willingness, frustrations and intentions on TB-IPC implementation (n=12).^15 16 34 40 50 53 66 68 81 94 96–98^ The influence of healthcare providers feeling appreciated and cared for on TB-IPC implementation was rooted in, for example, feelings of protection by management, neglect of primary care compared with secondary care and fear of accusations for own negligence when developing TB disease (n=12).^16 36 38 58 63 64 66 77 81 96 98 99^ Other studies reported patients’ and providers’ perceptions of patient unruly behaviour or non-compliance with TB-IPC (n=11).^16 36 39 47 53 58 74 77 80 94 101^ Some studies suggested the influence of staff’s sense of responsibility (n=7),^16 40 47 53 95 96 98^ for example, staff diverting TB-IPC responsibilities to the TB-IPC-trained person^53^ or to the individual.^16 95^

## Discussion

Research to date predominantly focuses on health system hardware, particularly human resources, and research with a primary aim to examine health system influences on TB-IPC implementation is underexplored. Our findings further reaffirm the interdependency between TB-IPC measures and the broader health systems within which they are implemented, and are in line with the WHO’s core components for IPC in healthcare contexts.^19^ However, using the Sheikh *et al*^28^ framework to critique the health system components proposed by the WHO, we note that these too predominantly fall within health system hardware.

Frequently reported hardware influences were: the availability, knowledge and educational development of staff; coordination and timeliness of diagnostics and service delivery; the availability of surgical masks and respirators; space for isolation and separation of patients with (presumptive) TB; TB-IPC IEC materials and tools; and funding. Less reported—than hardware—were software influences. These mostly referred to the correct and consistent use of TB-IPC measures, staff agency, and TB risk perceptions and fears. A considerably smaller share of studies explored the social, political and policy contexts within which TB-IPC is implemented. These mainly covered geographical or weather variations, TB stigma, the availability of facility-specific TB-IPC policies and monitoring, and evaluation of policy implementation.

Even within health system hardware domain there were neglected components. Few studies focused on training efforts for staff capacity to navigate opportunities and barriers in applying their knowledge or monitoring and feedback around TB-IPC implementation. Another underexamined area was the influence of knowledge and capacity of stakeholders not primarily located at health facility level, such as district IPC coordinators. Though our findings suggest that organisational coordination and supervision structures or mechanisms play a role in stimulating ownership of TB-IPC implementation and evaluation, it remains unexplored what processes, procedures and requirements are needed to establish and sustain such structures and mechanisms. It is also unclear to what extent stakeholders across the system are provided with decision space or are required to participate in the decision-making and implementation structures relevant to TB-IPC.

The functionality and maintenance of engineering controls as well as the availability and role of technology in support of appropriate TB-IPC implementation, such as CO_2_ monitors as a measure of adequate ventilation, were under-researched. Although we found emphasis on the influence of available space, its infrastructure and overall facility design, there is limited attention to the maintenance and augmentation of physical spaces. This suggests that clinic layout and renovations may be minimally emphasised in the planning, implementation and evaluation of TB-IPC interventions. Lastly, it is unclear how recording, communication and dissemination flows are or should be shaped in relation to TB-IPC, for example, what contributes to their sustainability and context sensitivity. We additionally note general limited detailing and insight into the financing of TB-IPC implementation.

We further need a deeper, more detailed understanding of what constitutes and contributes to the software influences identified and of how and by whom power and relationships are shaped. The influence of matters of legitimacy, authority and autonomy pervading hardware, software, policy and broader contextual influences remains largely underinvestigated. There is a dearth in knowledge on the wider social and political influences, interests, agendas and relationships at play. The depth and breadth of local and national policy documents, how and by whom these are formulated and prioritised as well as their transferability and applicability to local contexts were also underexamined. Given the inherent interconnectedness and permeability of each of these spheres to the other, as well as to the hardware sphere, our findings present a pivotal knowledge gap around the political economy and governance of TB-IPC implementation.

This review demonstrates that studies investigating TB-IPC implementation have relied mainly on fixed, structured assessments of practices and associated influences, which tend to be reductive in attempts to simplify or quantify. This significantly limits the analysis of the contextual complexities surrounding TB-IPC implementation. All studies lacked guidance by a conceptual framework, such as the Sheikh *et al*^28^ framework, to explore the system cross-cutting influences, processes and interactions. Subsequently, the identification of context-sensitive, cross-cutting and supportive implementation strategies to inform policy and practice is hindered, which can explain poor TB-IPC implementation. Thus, more flexible research design and holistic approaches to assessing and improving TB-IPC implementation policy and practice are required. Such approaches will help paint a complete picture, enable the identification of intervention areas and tailor responses to permeating complexities rather than individual influences.

### Whole system influences on TB-IPC implementation: a framework of permeability

**Figure 3:**
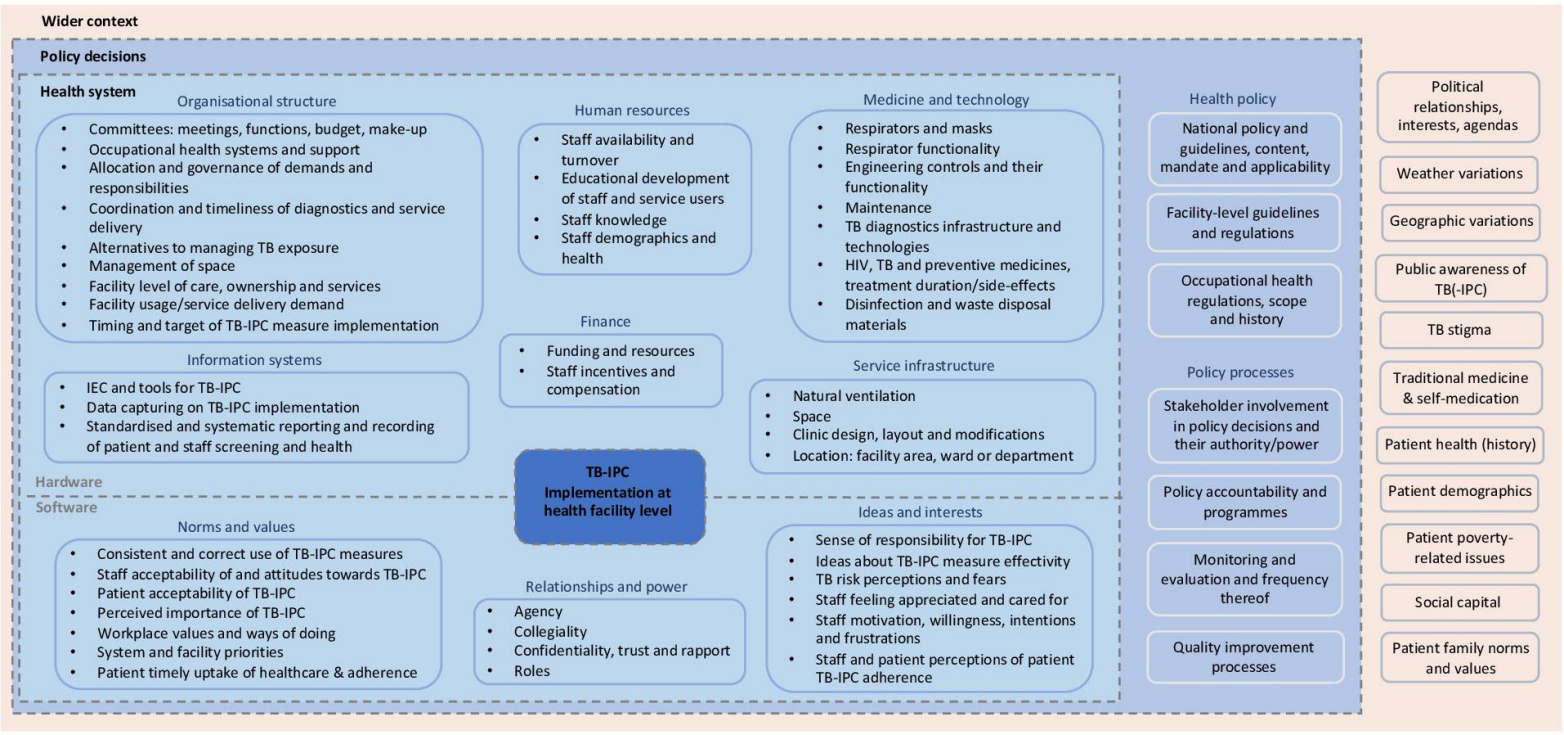
Whole system influences on TB-IPC implementation: a framework of permeability. IEC, information, education and communication; TB-IPC, tuberculosis infection prevention and control.

To guide future TB-IPC implementation research, practices and the development of supportive strategies that cut across the potential influences at play, we propose a whole system approach to TB-IPC based on our findings (figure 3). While synthesising the available evidence, we acknowledged a high level of heterogeneity in data obtained, as well as difficulties with meaningfully summarising and untangling health system influences. Adopting the Sheikh framework had two advantages. First, it allowed for the consideration of cross-level health system influences on TB-IPC. Second, it emphasised the interactions of system ‘software’ and ‘hardware’ elements that are at play and their permeability with respect to policy decisions and the sociopolitical context. Our adaptation of the framework suggests expansion of the social and political context to the ‘wider’ context, for example, to encompass weather and geographical location influences. We also stress the overarching permeability of—and inherent interactions between—the framework components and spheres as applied to TB-IPC implementation.

### Limitations

We recognise that there are papers that report and/or further illuminate some of the interactions between (1) the implementation of individual TB-IPC measures, (2) system influences, and (3) system influences and the implementation of (individual) TB-IPC measures. As such, we note diversity in relation to the depth and breadth to which influences were described and explained in included studies. This also included influences that were not predefined to be investigated at the outset of the study and emerged from data collection. Researcher bias and ambiguous descriptions of influences may have affected the conceptual organisation and interpretation of data, although this was done by two reviewers. Given our positionality as health system researchers, we acknowledge that researchers from other disciplines may have chosen other frameworks to inform analyses.

## Conclusion

Previous examinations of TB-IPC implementation at health facility level considered a wide variety of system and contextual influences, predominantly focusing on health system hardware. However, we need to adopt a whole system approach to (1) further investigate system cross-cutting influences and interactions that have bearing on the implementation of TB-IPC, with particular attention to health system software, policy processes and the wider context, and (2) develop strategies for improved and sustainable implementation of TB-IPC measures at health facility level.

## Data Availability

All data relevant to the study are included in the article or uploaded as supplemental information.
