# Supplementary material for: Health system influences on the implementation of tuberculosis infection prevention and control at health facilities in low-income and middle-income countries: a scoping review
Source: BMJ Glob Health. 2021 May 11;6(5):e004735. doi: 10.1136/bmjgh-2020-004735 (PMC8118012; doi:10.1136/bmjgh-2020-004735)

Supplementary Table 1: Database search strings as performed on 4 July 2018

Database	Search string	Results
Pubmed	((((((("tuberculosis") AND (("infection* control" OR "infection* prevention and control")))) AND (((((((("disease* transmission*" OR "infection* transmission*") AND "health facilit*")) OR "nosocomial") OR (("cross*infection*") AND "health facilit*")) OR "patient* to provider* transmission*") OR "disease* transmission* patient* to professional*" OR "infection* transmission* patient* to professional*") OR "disease* transmission* professional* to patient") OR (("patient* to patient* transmission*") OR "patient* to patient* cross infection*") OR "provider* to patient* transmission*" OR "disease* transmission* patient* to patient*" OR "infection* transmission* patient* to patient*")))))) OR (((tuberculosis[MeSH Terms]) AND disease transmission, infectious[MeSH Terms]) AND infection control[MeSH Terms])	649
CINAHL plus with full text (via EBSCOhost)	ditto	147
Medline	ditto	355
Web of Science (all databases)	TS=("tuberculosis" OR "tb") AND TS=("infection* control" OR "infection* prevention and control") AND TS=(("disease* transmission*" AND "health facilit*") OR ("infection* transmission*" AND "health facilit*") OR "nosocomial" OR ("cross*infection*" AND "health facilit*") OR "patient* to provider* transmission*" OR "disease* transmission* patient* to professional*" OR "infection* transmission* patient* to professional*" OR "disease* transmission* professional* to patient" OR "infection* transmission* professional* to patient" OR "patient* to patient* transmission*" OR "patient* to patient* cross infection*" OR provider* to patient* transmission* OR disease* transmission* patient* to patient* OR infection* transmission* patient* to patient*)	896
Scopus	((ABS ("tuberculosis") AND (ABS ("infection* control" OR "infection* prevention and control")))) AND ("disease* transmission*" AND "health facilit*") OR ("infection* transmission*" AND "health facilit*") OR "nosocomial" OR ("cross*infection*" AND "health facilit*") OR "patient* to provider* transmission*" OR "disease* transmission* patient* to professional*" OR "infection* transmission* patient* to professional*" OR "disease* transmission* professional* to patient" OR "infection* transmission* professional* to patient" OR "patient* to patient* transmission*" OR "patient* to patient* cross infection*" OR "provider* to patient* transmission*" OR "disease* transmission* patient* to patient*" OR "infection* transmission* patient* to patient*"	405

Supplementary Table 2: Post hoc inclusion and exclusion criteria

Inclusion criteria	Exclusion criteria
Empirical research and systematic literature reviews<br><br>Low and middle-income countries<br><br>All primary, secondary and tertiary health facilities that provide outpatient or temporary inpatient health services (including dental health facilities, clinical trial sites and mobile health facilities)<br><br>TB IPC measures (if not primary research focus, IPC measure implementation for the purpose of reducing TB transmission should be mentioned in the results section)<br><br>All quantitative, qualitative, mixed method, intervention and policy analysis studies of TBIPC implementation that describe practices or processes (observational studies) or introduce (additional) measures or socio-behavioural/organisational interventions.	Editorials, case tracing reports, non-systematic literature reviews, anecdotal/non-empirical descriptions, guidelines and recommendations.<br><br>Sole focus on high income countries<br><br>Health facilities such as elderly care homes, long term care facilities.<br><br>Sole focus on IPC for TB transmission via surgical/diagnostic tools, other airborne diseases, nonairborne diseases or other health care associated infections<br><br>Studies reporting prevalence or incidence of nosocomial TB only.<br><br>Studies evaluating TBIPC measures only in terms of risk, nosocomial incidence and prevalence outcomes and not in terms of nature or extent of their implementation.<br><br>Studies assessing the associations between physiological risk factors and nosocomial TB incidence.<br><br>Evidence on medical effectiveness of new therapies, diagnostics, surveillance, the optimisation of tools and their influence on the effectiveness of IPC measures or on nosocomial infection rates (technical advancements to improve effectiveness of individual IPC measures, but not their implementation)

Supplementary Table 3: Health systems influences on TB-IPC implementation at health facility level

Component	Item	Elements affecting TB-IPC implementation at health facility level
Social and political context (n=42) [5,14,16,17,35,36,38-41,43,46,47,50,51,53,56,58,60,61,63,65-67,69,71,72,74,77,80-85,88,90,92,93,96,98,99]		- Geographic variations, e.g. (sub-)district, province, rural/urban and gross domestic product (n=14) [5,40,41,51,53,56,63,67,69,83,84,90,92,96]<br>- Seasonal or circadian weather variations (n=12) [14,16,17,43,53,61,71,72,77,80,81,96]<br>- Stigmatisation of TB patients (n=9) [40,47,60,67,74,77,81,93,98]<br>- Patient current health or health history (n=6) [35,38,46,50,65,67]<br>- Patient demographics (n=6), [38,39,41,50,67,72] including gender, sex, age, income, education, employment status, marital status<br>- Public,[39] patient [39,67,74,82,85] or visitor/family/carers [74,82] awareness of prevention measures and TB (n=5)<br>- Social capital (n=4): [,36,66,77,98]<br>○ People important to health providers wanting the health providers to protect themselves against TB [66]<br>○ Family members who are financially dependent on healthcare providers [77] or TB patients [36]<br>○ Family support for TB patients [98]<br>- Patient poverty-related issues (n=3): [36,74,98]<br>○ Patient job insecurity [36]<br>○ Availability of a social grant for poor and moribund TB patients (on treatment adherence) [74]<br>○ Patient's lack of money for transport to health facility, nutrition, food availability, and drugs and substance abuse [98]<br>○ Living in overcrowded and impoverished conditions [74,98]<br>- The influence of patient family values and norms on TB-IPC implementation (n=2): [36,98]<br>○ Family mealtime customs [36]<br>○ Family scrutinising health provider when leaving patient alone to get protective equipment [98]<br>- Traditional medicine and self-medication (n=2): [74,99]<br>○ The role of traditional healers [74]<br>○ Self-medication and home remedies, [99] and traditional medicine [74]<br>- Political relationships, interests and agendas (n=2) [83,98]<br>○ Deepening of global economic relationships [83]<br>○ Political commitment to TB-IPC and occupational health [83]<br>○ Ministry of Health initiated TB health education campaigns [98]

		○ Prioritisation of maternal and infant health indicators over TB incidence by Ministry of Health [98]
**Policy decisions** (n=34) 5,15-17,32,36,37,40,42,43,47,49,50,52,59,62–64,16,68,69,72,74,78–84,86-88,90,91,93,94,102	**Health Policy** (n=32) 5,15-17,36,37,40,42,43,47,49,50,62–64,69,72,74,78–84,86,88,90,91,93,94,102	- Availability of a facility-level (TB-)IPC plan (n=21) [5,17,36,37,40,42,43,47,50,62,64,69,72,84,86,88,90,91,93,94,102]<br>○ Facility-level TB-IPC plan (n=14) [40,43,47,50,62,64,69,84,86,90,91,93,94,102]<br>○ Facility-level IPC plan (n=7) [5,17,36,37,42,72,88]<br>○ Content more generally (n=6) [5,37,43,88,90,94]<br>○ Content more specifically:<br>▪ Whether and IPC plan included TB (n=1), [37] TB control (n=1), [72] airborne related activities (n=1) [88]<br>▪ What guidelines TB-IPC plans are based on and whether this is up to date (n=1) [90]<br>▪ Whether the IPC plan contained a statement of endorsement by the facility manager (n=1) [43]<br>- Availability of national-level IPC policies and guidelines (n=14),[16,37,42,43,49,50,74,78–81,91,93,94] and/or their:<br>○ Applicability (n=3) [15,81,83]<br>○ Content (n=1) [83]<br>○ Incorporation of HCW protection (n=1) [83]<br>○ Alignment with international practices (n=1) [83]<br>○ Mandate (n=1) [83]<br>- Availability of more specific regulations related to (n=13): [5,15,17,42,50,63,64,78,82,84,91,93,102]<br>○ Caring for/assisting patients (n=4) [42,64,84,91]<br>○ Staff screening (n=4) [15,63,78,91]<br>○ Masks (n=3) [5,17,102]<br>○ Visitors (n=2) [64,82]<br>○ TB patient isolation (n=1) [5]<br>○ HCW training (n=1) [64]<br>○ Environmental controls (n=2) [64,91]<br>○ Monitoring of TB IC regulations in high risk areas (n=1) [42]<br>○ Staff redeployment (n=1) [82]<br>○ Monitoring average waiting times (n=1) [42]<br>○ Separation of (presumptive) infectious patients or coughing patients (n=1) [93]<br>○ Cough etiquette for patients (n=1) [93]<br>○ HIV/AIDS (n=1) [50]<br>- Availability of occupational health regulations (n=6),[5,17,78,79,83,94] and/or looking at:<br>○ History (n=1) [83]

		○ Scope (n=1) [83,94]
	**Policy processes** (n=26)<br>5,16,32,36,42,43,47,52,59,62,63,68,78–81,83,84,86-88,90,91,93,94,102	- Monitoring and evaluation of policy implementation (n=17),[5,16,36,42,43,47,62,63,78–81,83,84,88,91,93] and frequency (n=7) [42,43,78,84,88,91,93]<br>- Quality improvement processes (n=10) [42,43,59,68,79,86,88,91,93,102]<br>○ Intervention studies (n=6) [42,59,68,79,88,93]<br>○ Regarding clinic layout and ventilation (n=4) [43,86,91,102]<br>- Stakeholder involvement in policy development processes: (n=7) [42,59,81,83,87,90,94]<br>○ Health providers (n=4) [42,59,81,94]<br>○ Experts (n=3) [83,90,94]<br>○ IPC committee members (n=1) [87]<br>○ Authority of stakeholders involved (n=1) [83]<br>- Policy accountability (n=7) [32,42,43,52,80,81,83]<br>○ Availability of TB-IPC programmes (n=2) [32,52]<br>○ Availability of occupational health programmes (n=2) [42,43]<br>○ Past lawsuits in which HCWs sued hospitals for compensation for occupational TB (n=1) [83]<br>○ Health providers' perceived inability to prove occupationally acquired TB: "TB is everywhere" (n=2) [80,81]
**The health system**		"The health(care) system" (n=2) [36,72]
**System hardware** (n=77)<br>5,11,14–17,32–102	**Human resources** (n=66)<br>5,11,15–17,32,33,36–37,39,43–60,62–72,74–96,98–102	Educational development (n=45) [16,17,32,36,37,39,41–44,47,49,50,52–55,58–60,62–64,67,69–72,74,78–82,84,86–89,91,93,94,96,98100,101]<br>- Availability of or participation in staff training on (n=38): [16,17,36,41,43,44,47,49,50,52,53,55,58–60,62–64,70–72,74,78–82,84,86–89,91,93,94,96,98,101]<br>○ (TB) IPC (n=31) [36,41,43,44,47,49,50,52,53,55,60,62–64,70,72,74,79–82,84,86–89,93,94,96,98,101]<br>▪ In particular on respirators (n=12) [16,17,43,53,59,62,0,71,81,88,91,96]<br>○ TB (n=7) [52,53,58,62,64,91,94]<br>○ Training in OH (n=2) [78,79]<br>○ Assessment, incident investigation, and use of information/data capturing system (n=1) [79]<br>○ On the use of an information system (n=1) [59]<br>○ HIV stigma, patient violence, health and safety (n=1) [79]<br>○ Psychiatry (n=1) [58]<br>○ With more specific focus on training characteristics: (n=36) [17,36,37,42,43,44,54,55,58,62–64,70,72,74,79,81,82,84,86–89,91,93,94,96,99,101]<br>▪ Type of training: (n=20) [17,33,36,42,43,58,70,72,74,79,81,82,84,88,89,91,93,94,96,98]<br>• In-service/continuing training (n=9) [17,36,58,74,81,84,93,96,98]

		• Pre-employment/orientation (n= 6) [70,82,84,89,91,96]<br>• Hands-on TB-IPC training programme or mentoring (n=4) [33,42,79,81]<br>• Training of trainers (n=4) [42,43,93,94]<br>• Training video (n=2) [42,43]<br>• Regional training (n=2) [93,94]<br>• Organisation, content and resources provided by training programme (n=2) [79,93]<br>• TB campaigns (n=1) [99]<br>• Developing a TB IC template (n=1) [94]<br>• Meetings with facility doctors (n=1) [58]<br>• International training followed by in-country training of architects and engineers (n=1) [88]<br>• Mandatory (n=1) [72]<br>• Refresher courses (n=1) [81]<br>• Exchanging international experiences (n=1) [79]<br>▪ Target (n=15) [36,37,43,64,72,74,79,81,86,88,91,93,94,98,101]<br>▪ Frequency (n=15) [37,43,44,54,55,62–64,72,81,88,91,93,96,98]<br>▪ Duration (n=3) [33,54,93]<br>▪ Adequacy/specificity (n=1) [64]<br>- Community TB, IPC or health education (n=21) [32,36,37,39,43,44,47,52,53,62,67,69,72,80–82,84,91,93,98,100]<br><br><u>Knowledge</u> (n=40) [11,15,16,32,40,42,44,49,50,53–55,58,59,63–64,66,68,70,72,74,75,80–83,85-87,89,91,93–96,98,99,101]<br>- Staff knowledge of (n=38): [11,15,16,32,40,42,44,49,50,53–55,58,59,62–64,66,68,70,72,74,75,80–83,85,86,87,89,91,93–96,98,99,101]<br>○ Specific TB-IPC measures (n=28) [11,16,40,42,44,49,53,54,59,62–64,66,68,72,74,75,81–83,85,89,93–96,101]<br>○ TB infection, disease and treatment (n=16) [11,15,16,44,55,58,64,66,68,74,89,91,95,96,98,99]<br>○ TB transmission (n=11) [11,15,16,32,55,58,66,74,89,95,96]<br>○ TB risk factors/susceptibility (n=7) [11,15,50,55,66,86,96]<br>○ IPC related Policy (n=9) [16,53,68,70,74,80,81,87,99]<br>○ Risk of TB (n=5) [15,58,59,85,99]<br>○ Epidemiology (n=2) [93,99]<br>○ Patient context (n=1) [98]<br>○ Research/program implementation (n=1) [68]<br>○ Writing and computer skills (n=1) [68]

		Availability of staff (n=36) [5,16,17,32,40,42-44,47,49–53,57,58,62,64,69,72,74,76,78–82,84,87,88,90,91,93,94,98,102]<br>- General staff availability (n=19),[5,40,43,47,49,52,53,58,74,79–82,84,90,91,93,94,98]<br>○ Staff turnover (n=3) [57,82,91]<br>○ Staff numbers (n=6) [5,40,52,84,90,91]<br>- Availability of staff responsible for IPC (n=19): [16,17,40,42,43,44,50,51,53,57,58,64,69,76,78,84,88,93,102]<br>○ The IPC focal person's qualification (n=3) [50,58,78], time (n=2) [64,78] and continuity (n=1) [88] on the job.<br>- Availability of staff for TB-IPC specifically (n=7) [43,52,69,72,76,87,90]<br>- Availability of staff with other specific roles: (n=14) [5,17,32,42,43,53,62,64,78,81,82,88,91,98]<br>○ Environmental control engineering staff (n=4) [5,53,88,91]<br>○ Laboratory staff (n=4) [17,32,53,82]<br>○ Dedicated staff to open windows (n=3) [62,81,88] and vents (n=1) [88]<br>○ occupational health nurse (n=2) [64,78]<br>○ TB nurse (n=2) [17,62]<br>○ Cough monitor (n=2) [42,43]<br>○ Health educator (n=1) [98]<br><br>- Healthcare provider demographics (n=19) [15,36,44,45,49,50,53–55,57,60,63,64,70,79,80,89,92,96] such as gender or sex, age, job category/cadre, years of experience, employment status and level of education<br>- Healthcare provider health (n=5): [5,58,66,81,96]<br>○ Having a cold, flu or rhinitis (n=2) [58,96]<br>○ TB infection or death among colleagues (n=4) [5,66,81,96]
	**Organisational structure** (n=61)<br><br>[14-17,32,34–43,44,47,49–53,55,57–61,62,64,66,68,69,71–84,88–94,96,98–101]	- Coordination and timeliness of diagnostics and service delivery (n=33) [16,17,32,34,35,39–43,47,50,52,53,59–61,62,66,68,72-74,79–82,91,92,94,98,100,101]<br>- Allocation, uptake and governance of demands and responsibilities (n=27) [36,40,43,47,49,50,53,57,58,60,62,66,68,74,76,78–83,88,91,93,94,96,98]<br>- Occupational health systems and support (n=26) [14,34,36,42-44,47,50,51,57,58,64,66,69,72,77,78,80,81,83,84,88,91,93,94,98]<br>- Timing/point (e.g. at facility entry) and target (e.g. staff, visitors and patients) of TB-IPC measure implementation (n=19) [16,37,40,43,44,47,50,53,55,62,64,66,69,72,74,80,82,99,100]<br>- Inter-facility differences (n=18), [15,38,40-42,47,50-53,57,66,76,78,84,90,92,96] including more specifically:<br>○ Level of care (n=9) [15,40,50–52,57,84,90,92]<br>○ Ownership (n= 6) [40,41,47,50–52]<br>○ Service availability (n=4) [15,42,51,52]

		- Management of how space is used (n=17) [14,17,36,40,47,50,53,72,74,75,77,79–81,96,98,101]<br>- Facility usage/service delivery demand (n=12): [17,38,40,50,52,77,83,84,91,92,98,99]<br>▪ Patient load (n=12) [17,38,40,50,52,77,83,84,91,92,98,99]<br>▪ Visitor numbers (n=1) [52]<br>- Committees (n=11): [17,36,47,62,72,76,78,82,84,93]<br>○ IPC committee existence [17,47,58,62,72,76,78,82,84,93]<br>▪ Meetings (n=5) [17,62,72,82,88]<br>▪ Allocated functions (n=2) [76,82]<br>▪ Make-up (n=2) [72,82]<br>▪ Allocated budget (n=1) [72]<br>○ Availability of a health and safety committee (n=1) [78]<br>- Alternatives to managing exposure to TB patients (n=11) [32,37,58,64,71,73–75,80,89,100]
	**Medicine and technology** (n=52)<br><br>5,11,14–17,36,37,40,42-4,447,49,50,52,53,55,56,58,60–64,66,69–72,74,75,77–84,86,88,91–94,96,98–102	- Availability of masks/respirators for service users and providers (n=40), for: [5,14–17,36,40,42-44,47,49,52,53,55,60,62–64,66,69,70,72,74,75,77,79–82,84,86,88,91,93,96,98–100,102]<br>○ Staff (n= 31) [5,14–17,36,40,43,44,49,52,53,60,62,63,66,70,72,74,77,80–82,84,86,91,93,96,98,99,102]<br>○ Patients (n=26) [5,14,16,40,43,47,53,55,60,62,63,66,69,72,74,75,79,81,84,86,88,91,93,98,99,102]<br>○ Visitors (n=1) [64]<br>- Respirator functionality (n=24): [11,16,17,36,37,42,49,53,58,60,62,64,66,72,77,78,80,81,84,93,96,98,99,102]<br>○ Design (n=13) [11,16,36,49,53,58,60,64,66,77,80,81,96]<br>○ Fit testing (n=11) [17,42,62,64,72,80,81,84,93,96,102]<br>○ Quality (n=4) [64,78,93,96]<br>○ Cost (n=4) [80,96,98,99]<br>○ Maintenance (n=2) [37,96]<br>- Availability of "engineering controls" (including mechanical ventilation, UVGI, airflow control) (n=25) [16,36,42,43,44,47,49,50,53,56,58,62,64,69,71,72,74,77,80,81,83,84,92,94,102]<br>- Availability of hand hygiene, cleaning and/or waste disposal materials (n=20) [16,40,42,43,47,49,55,61–64,66,72,75,79,81,82,88,93,102]<br>- Availability of personal protective equipment (n=15) [36,42,49,56,58,61,64,74,77–79,81,93,101,102]<br>- Maintenance in general (n=11),[5,14,43,53,56,71,72,77,80,81,94] and more specifically of:<br>○ UVGI lights (n=5) [14,43,72,77,81]<br>○ Extractor fans (n=2) [43,72]<br>○ HEPA filters (n=1) [72]<br>- Functionality of engineering controls (n=7),[63,64,72,77,80,81,94] more specifically of:

		○ Fans (n=5) [63,64,72,77,80]<br>○ UVGI lights (n=3) [72,77,81]<br>- Availability of infrastructure and technologies for TB diagnostics (n=8) [17,40,44,52,55,56,58,92]<br>- Availability of TB medicines, HIV treatment or IPT (n=4) [42,44,55,72]<br>- Side-effects and duration of TB treatment course (n=2) [74,98]<br>- Availability of equipment to keep patients warm when windows are opened (n=1) [66]
	**Service infrastructure** (n=46)<br>5,14,16,17,33,36,37,40,42-44,47,50,52,53,55,56,58,61,62,64,66,69,71-75,77,79–82,84,86-88,91–94,96,98,100–102	- Space for (presumptive) TB patient isolation and separation (n=31) [5,16,17,40,42,43,44,47,50,52,53,55,56,61–64,72,74,75,80,81,83,84,88,91,92,98,100–102]<br>- Present infrastructure and clinic design for appropriate natural ventilation (n=26) [17,36,37,40,42,43,50,53,56,62,66,69,71,72,74,77,79–82,86,88,91,98,100,102]<br>- Facility building structure (n=22) [17,40,43,47,50,53,55,56,58,62,69,72,75,79–82,84,93,94,100,101]<br>○ Facility layout [55,58,62,69,72,75,82,84]<br>○ Modifications.[40,43,47,53,72,75,82]<br>- Location: facility area, ward or department (n=22) [14,16,17,33,37,40,44,47,50,53,61,62,64,73,74,79,82,84,87,88,96,100]<br>- Availability of adequate space (n=11) [17,36,40,47,50,56,77,79,81,94,101]<br>- Reliability of electricity (n=1) [72]
	**Information systems** (n=31)<br>5,17,32,38,42,43,47,49,50,52,53,59,62–64,69,71–74,78,79,81–83,88,90,91,93,94,97,102	- Information, education and communication materials and tools for TB-IPC (n=21) [42,43,47,49,50,53,59,62–64,72,74,79,81–83,88,91,93,97,102]<br>- Standardised and systematic record keeping and reporting of patient and staff screening and TB diagnosis (n=16) [17,32,38,42,43,50,52,59,69,72,73,78,81,83,90,94]<br>- Data capturing on TB-IPC implementation (n=14) [5,32,43,53,62,63,69,71,72,79,81,83,88]
	**Financing** (n=27)<br>11,16,38,40,42,43,47,49,53,58,62–64,66,68,77,79–83,88,93,94,96,98,99	- Availability of funding or resources (n=21) [16,38,40,47,49,53,58,62–64,66,68,79–81,88,93,94,96,98,99]<br>- Incentives and compensation for staff (n=8) [58,64,77,80–83,98]<br>- Funding specifically for (n=4): [11,42,43,94]<br>○ Maintenance (n=1) [94]<br>○ Equipment/supplies (n=3) [11,42,43]
**System software** (n=56)	**Norms and values** (n=52)	- Correct and consistent use of TB-IPC measures (n=39) [14–17,34,37–42,47,49,50,53,55,58,61,63,64,66,68–72,74,75,77,79,80,82,84,88,93,94,98–100]<br>- Workplace values and ways of doing (n=24) [16,36,40,43,53,57,58,60,66,72,74,75,77–82,93,94,96,98,99,101]

11,14–17,34,36-44,47,49,50,53,55–58,60-72,74,75,77–84,86,88,93,94,96–101	11,14-17,34,36-43,47,49,50,53,55,57,58,60-72,74,75,77–82,84,88,93,94,96–101	- Staff acceptability of and attitudes towards TB-IPC practices (n=22) [11,15,39,40,43,50,53,57,62–64,66,68,70,74,78–82,93,96]<br>- Perceived importance of TB-IPC by staff and patients (n=19) [15,16,36,39,50,53,55,58,65–68,80,81,94,96,97,99,101]<br>- Patient acceptability of TB-IPC measures (n=14) [16,36,39,40,53,60,64–67,77,81,98,101]<br>- System and facility priorities (n=9) [36,42,47,49,53,94,96,98,101]<br>- Patient non-adherence to TB treatment (n=4) [36,60,67,74]<br>- Late uptake of hospital care by patients or their guardians (n=2) [74,99]
	**Relationships and power** (n=33)<br>16,17,36,39,40,42-44,47,49,53,56–58,60,62,64,66,68,72,74,75,77,78,80–82,86,93,94,98,99,101	- Elements contributing to agency to implement TB-IPC (n=26), of: [17,36,39,40,42-44,47,57,58,60,64,66,68,72,74,75,78,80–82,86,93,94,98,101]<br>○ Staff (n=21) [36,40,42,43,44,47,58,60,66,68,72,74,75,78,80,81,86,93,94,98,101]<br>○ Staff with specific IPC responsibilities (n=3) [64,78,94]<br>○ IPC committees (n=2) [17,82]<br>○ Patients (n=2) [39,98]<br>- Collegiality among staff and between patients and staff (n=15) [36,40,44,49,58,62,66,68,72,74,77,81,82,93,98]<br>- Confidentiality, trust and rapport among staff and between patients and staff(n=15) [16,36,40,44,53,56,57,60,66,68,74,78,81,94,99]<br>- Roles of system actors (n=4) [40,47,81,98]
	**Ideas and interests** (n=31)<br>15,16,34,36,38-40,47,49,50,53,55,58,60,63,64,66–68,74,77,80–83,94,96–99,101	- TB risk perceptions and fears (n=23) [15,16,36,39,40,49,55,58,60,63,64,66,74,77,80–83,96–99,101]<br>- Ideas about TB-IPC measure effectivity (n=16) [16,36,55,58,64,67,74,77,80,81,83,96–99,101]<br>- Staff motivation, willingness, frustrations and intentions (n=12) [15,16,34,40,50,53,66,68,81,94,96-98]<br>- Health providers feeling appreciated and cared for (n=12) [16,36,38,58,63,64,66,77,81,96,98,99]<br>- Patient and staff perceptions of patient unruly behaviour or non-compliance with TB-IPC (n=11) [16,36,39,47,53,58,74,77,80,94,101]<br>- Staff sense of responsibility for TB-IPC (n=7) [16,40,47,53,95,96,98]

Supplementary Figure: Health policy and systems perspectives. Adopted from Sheikh et al. (2011) with permission.[30]

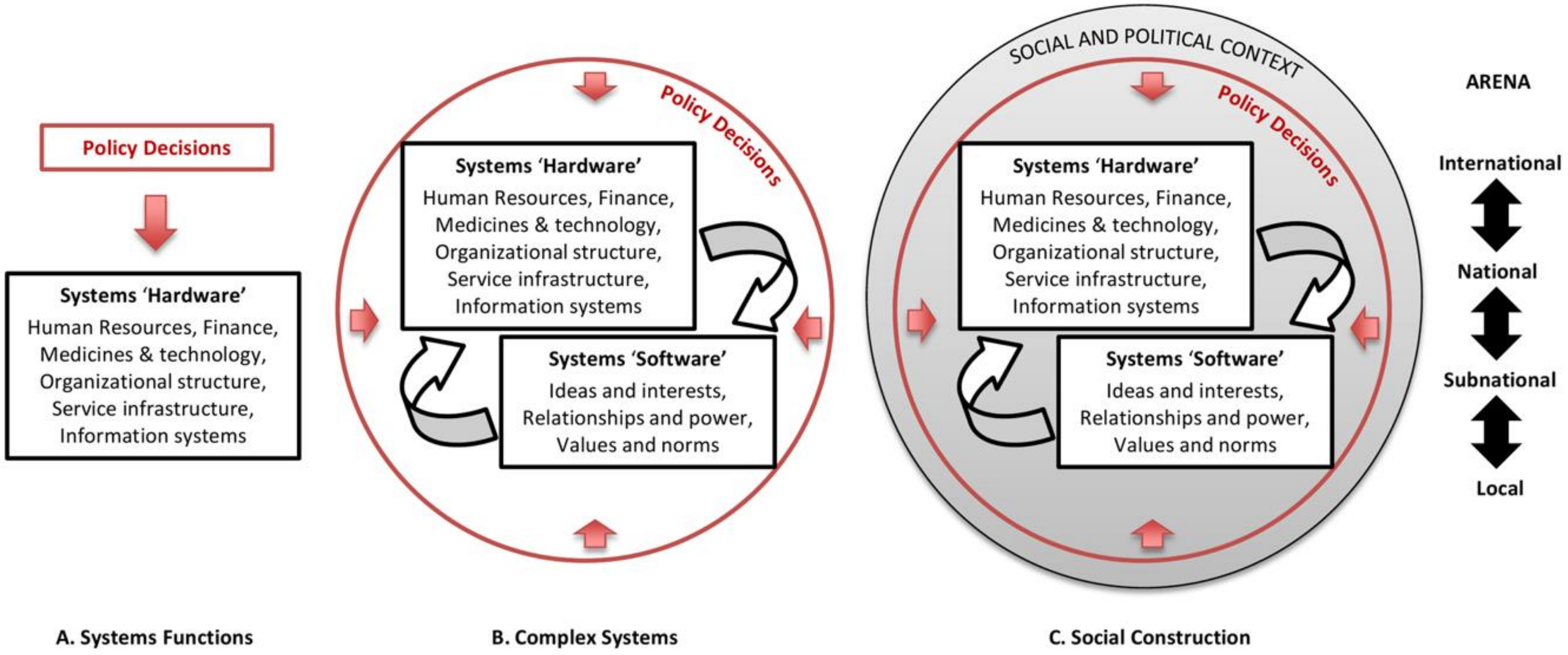

Supplement: Supplementary data [file bmjgh-2020-004735supp001.pdf]
