# Supplementary material for: Health system influences on the implementation of tuberculosis infection prevention and control at health facilities in low-income and middle-income countries: a scoping review
Source: BMJ Glob Health. 2021 May 11;6(5):e004735. doi: 10.1136/bmjgh-2020-004735 (PMC8118012; doi:10.1136/bmjgh-2020-004735)
Supplement: Supplementary data [file bmjgh-2020-004735supp002.pdf]

Supplementary Data Extraction Form

**Extract**	**Operational guidelines**
First Author	Surname only
Year of publication	
Title	
Study aim	In-text, and in cases where this was less clear taken from abstract.
Country	
Level of care	Primary health facilities (including (community) health centres and clinics, such as specifically for dentistry, HIV or TB) or hospitals (including "hospitals", district, regional, secondary and tertiary health facilities), or both.
Study type	Observational or experimental/intervention study.
Methodology	Quantitative, qualitative or mixed methods: If not clear from methods adopted, this categorisation was based on type of analysis. If that was also not clear, the decision was based on the way in which data is presented (numerical, non-numerical or a combination.
Data collection methods	Relevant excerpts copied for categorisation.
Questionnaires	Including checklists, assessment/audit tools, self-administered or interview-administered, questionnaires with either/both open- or closed-ended questions and structured interviews.
Focus group discussions	Including group interviews, participatory workshops.
Interviews	Not to support implementation of a questionnaire, but as an alone-standing method - these are semi-structured/in-depth or unspecified but not structured.
Observations	Unstructured observations or unspecified.
Existing document/record review	Includes patient and health provider records, facility-level policy and guidelines.
Diagnostics	Any diagnostic tests done as part of the study.
Ventilation measurements	Includes airflow measurements and microbial air samples.
(Inter)national policy review	
Participants included	Providers, patients, both or N/A. If only general audit tool was used this is not applicable (N/A).
Intervention	If intervention study, include extracts of intervention descriptions.
Any influences on TB-IPC implementation at health facility level	Full quotations or descriptive summaries presenting contextual and health system influences as reported in results sections, including system actor characteristics.
